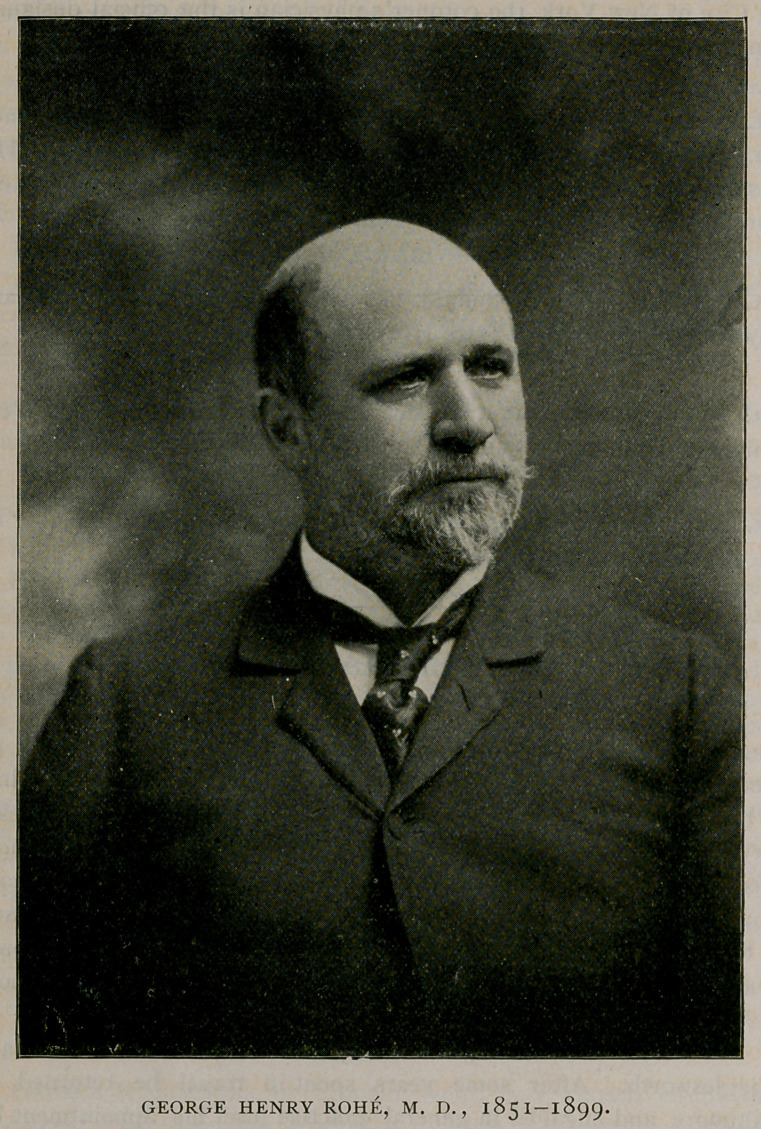# Dr. George Henry Rohé

**Published:** 1899-03

**Authors:** 


### OBITUARY.

Dr. George Henry Rohé, of Baltimore, Md., died suddenly at New Orleans, La., February 6, 1899, aged 48 years. At the time of his death he was in attendance upon the National Prison Congress, serving as a delegate from Maryland by appointment of the Governor. His parents were natives of Bavaria, but he was born in Baltimore county, January 26, 1851. His preliminary education was obtained in the public and parish schools of Baltimore and his professional studies were carried forward at the University of Maryland School of Medicine, from which he graduated in March, 1873.

He afterward studied dermatology in Boston under Dr. Edward Wigglesworth. After some years spent in travel he returned to Baltimore and engaged in general practice until his appointment by Mayor Davidson as city health commissioner in 1890. For a short time in 1885, he was acting assistant surgeon in the United States Army. He had been professor in the College of Physicians and Surgeons since 1881 and at different times had filled the chairs of therapeutics, materia medica, hygiene and mental diseases. He was appointed superintendent of Spring Grove Hospital for the Insane in

GEORGE HENRY ROHÉ, M. D., 1851–1899.

1891, and in 1896 was elected to take charge of the new hospital at Springfield, near Sykesville.

Dr. Rohé was a member of the American Medical Association, American Public Health Association, of which he was president when he died; American Association of Obstetricians and Gynecologists, and its president in 1894; the Medical and Chirurgical Faculty of Maryland, and its president in 1893; the American Medico-Psychological Association; American Electro-therapeutic Association; Clinical Society of Maryland; Baltimore Medical Association; Baltimore Neurological Society; Medical and Surgical Society of Baltimore; Southern Surgical and Gynecological Association; was a member of the committee on organisation of the first Pan-American Congress; American Academy of Political and Social Science; foreign associate member of the Societé Francaise d'Hygiene; and secretary and treasurer of the Rush monument committee. In 1894 he was elected an honorary member of the Society of Mental Medicine of Belgium and corresponding member of the Medico-Psychological Society of Paris.

His membership in all these several societies was conferred by reason of his special fitness for the same and was not in any sense a perfunctory honor. It indicates in a measure the thorough equipment of the man in many directions, but probably his greatest claim to fame is through the establishment of the second Maryland Hospital for the Insane, at Springfield. It was largely through his influence that the old Patterson estate, near Sykesville, was purchased of Ex-governor Frank Brown, which is as near an ideal location as can be found anywhere. The buildings are of the most approved architecture, and the system of management employed is the most humane that can be devised. Dr. Rohé was an alienist of the most advanced ideas in theory and practice and nothing that could be obtained or constructed was omitted in the care and treatment of persons suffering from mental disease under his care. His studies into the relationship between mental and pelvic diseases, and his advocacy of abdominal section, in appropriate cases, for the relief of insane women, identified his name with this advanced method, and his papers on the subject have been quoted all over the world. Dr. Rohé was also an author of conspicuous ability as well as versatility. He published a text-book of Hygiene, which ran through three editions; a work on Practical electricity in medicine and surgery, and a Manual of skin diseases. He was associate editor of the Annual of the Universal Medical Sciences, and published many monographs, relating to the several branches of medicine with which his name has been identified.

The news of his sudden death, as published in the press dispatches next day, spread a gloom amongst his many friends in all sections of the country, and the shock was appalling to those who knew him intimately.

Dr. Rohé was one of the best known physicians not only in Baltimore and the State of Maryland, but all over the country. He was not only gifted in his profession, but possessed all the charms belonging to a man of culture, refinement, amiability, and strength of character. Socially, he had but few equals as a host or entertainer and his tastes led him to often serve in those relations. His door was always open to his friends and his kindness of heart and sympathy with the distressed made him a liberal giver to those deserving of charity. It is difficult within the limitations of such a notice to do justice to such a memory, especially when the writer, as in the present instance, is overwhelmed with his personal loss.

Dr. Rohé was married in 1890 to Miss Mary Lauderman Coffin, of Baltimore, a descendant of Tristam Coffin, the original settler of Nantucket Island in 1660. He is survived by his wife and one child, Margaret Rohé—Gretchen, as she is best known to her father's intimate friends. They, widow and daughter, receive the tender sympathy of a multitude of people.

## Figures and Tables

**Figure f1:**